# Distribution and pathogen prevalence of field-collected ticks from south-western Korea: a study from 2019 to 2022

**DOI:** 10.1038/s41598-024-61126-y

**Published:** 2024-05-29

**Authors:** Kwang gon Kim, Da jeong Hwang, Jung wook Park, Mi geum Ryu, Yujin Kim, So-Jin Yang, Ji-Eun Lee, Gi seong Lee, Ju Hye Lee, Ji sun Park, Jung mi Seo, Sun-hee Kim

**Affiliations:** Division of Infectious Disease Investigation, Health and Environment Research Institute of Gwangju, 584 Mugindae-ro, Seo-gu, Gwangju, 61954 Republic of Korea

**Keywords:** Climate-change ecology, Diseases

## Abstract

Hard ticks are known vectors of various pathogens, including the severe fever with thrombocytopenia syndrome virus, *Rickettsia* spp., *Coxiella burnetii*, *Borrelia* spp., *Anaplasma phagocytophilum,* and *Ehrlichia* spp. This study aims to investigate the distribution and prevalence of tick-borne pathogens in southwestern Korea from 2019 to 2022. A total of 13,280 ticks were collected during the study period, with *H. longicornis* accounting for 86.1% of the collected ticks. *H. flava, I. nipponensis* and *A. testudinarium* comprised 9.4%, 3.6%, and 0.8% of the ticks, respectively. Among 983 pools tested, *Rickettsia* spp. (216 pools, 1.6% MIR) were the most prevalent pathogens across all tick species, with *R. japonica* and *R. monacensis* frequently detected in *I. nipponensis* and *Haemaphysalis* spp., respectively. *Borrelia* spp. (28 pools, 0.2% MIR) were predominantly detected in *I. nipponensis* (27 pools, 13.8% MIR, P < 0.001). Co-infections, mainly involving *Rickettsia monacensis* and *Borrelia afzelii,* were detected in *I. nipponensis.* Notably, this study identified *R. monacensis* for the first time in *A. testudinarium* in South Korea. These findings offer valuable insights into the tick population and associated pathogens in the region, underscoring the importance of tick-borne disease surveillance and prevention measures.

## Introduction

Hard ticks are a type of external parasite that feeds on the blood of both animals and humans and are known to transmit various viruses, parasites, and bacteria^1^. In a study on geographical distribution of Ixodid ticks in Korea from 2013 to 2015, *Haemaphysalis longicornis* accounted for 88.9%, followed by *H. flava* (10.1%), *Ixodes nipponensis* (0.5%), *I. persulcatus* (0.2%), *H. japonica* (0.2%), *Amblyomma testudinarium* (0.1%), and *I. granulatus* (< 0.1%)^2^. *H. longicornis* peaked in May to July (with larvae in September, and nymphs in May, and adults in July), while *H. flava* collected mainly in September to October (with larvae and adults in September, and nymphs in October) based on dry-ice bait trap method.^2^. Hard ticks mainly found in the southern region of Korea include *H. longicornis*, *H. flava*, *I. nipponensis*, and *A. testudinarium*^3^.

The important tick-borne pathogens (TBPs) that are transmitted by hard ticks include the following: Severe Fever with Thrombocytopenia Syndrome (SFTS) virus, *Rickettsia* spp., *Coxiella burnetii*, *Borrelia* spp., *Anaplasma phagocytophilum, Ehrlichia* spp^1,4^. SFTS is a vector-borne infectious disease that was first reported in China in 2011^5^. Its incidence has increased in China, Japan, and Korea, and it is currently designated as a category three national notifiable infectious disease in Korea^6^. It is mainly transmitted by *H. longicornis*, with *H. flava*, *I. nipponensis*, and *A. testudinarium* also known as vectors for the SFTS virus in Korea^7^. The prevalence of SFTS virus in ticks^8^, and wild animals^9–11^ has been determined. Kim et al*.* also reported a molecular epidemiological correlation between a patient with SFTS and questing ticks collected from the patient's residence^12^.

Spotted fever group rickettsioses (SFGR) are febrile diseases caused by *Rickettsia* species associated with chiggers, fleas, and hard ticks^13,14^. In Korea, *R. rickettsii* and *R. japonica* were confirmed in *H. longicornis* by PCR^13^. Japanese spotted fever (caused by *R. japonica*) was first reported in 2005^14^. Moreover, *R. monacensis* was also isolated from a patient in Korea^13^. Recently, a study provided the first description of *R.raoultii* detected in *H. longicornis* ticks, which were collected from patients with a history of tick bites in Korea^15^. This pathogen was also shown to have a high prevalence in ticks collected from dogs in Korea^16^.

Q fever is a globally occurring zoonotic illness caused by *Coxiella burnetii*^17^. *C. burnetii* is known to be transmitted to humans via inhalation of contaminated aerosols from animals and consumption of contaminated milk^18^. The role of *C. burnetii*-infected ticks in human Q fever is being disputed, as ticks are not essential vectors for *C. burnetii* transmission. However, several studies have shown ticks may play an important role in the transmission of coxiellosis between livestock and wildlife, which may lead to human coxiellosis^19^.

Lyme disease is a tick-borne illness caused by *Borrelia burgdorferi* sensu lato (s.l.), which comprises approximately 20 genospecies^20,21^. In Korea, *Borrelia burgdorferi* s.l. was first detected in *Ixodes* ticks in 1993, and the first human case of Lyme disease was reported in the same year^22^. To date, *Borrelia afzelii*, *B. garinii, B. tanukii, B. turdi, B. yangtzensis, B. bavariensis*, and *B. valaisiana* genospecies have been identified in ticks and wild animals in Korea^21^.

*Anaplasma phagocytophilum* and *Ehrlichia* spp. belong to the family *Anaplasmataceae* and share similar characteristics^23^. Both *A. phagocytophilum* and *Ehrlichia spp.* have been identified in *H. longicornis, I. nipponensis,* and *I. persulcatus* in Korea^13^.

As a result of tropical climate change, the summer season in South Korea is becoming longer and warmer^24,25^. This leads to an increased risk of tick-borne infectious diseases due to the higher survival rate, increased egg-laying rate, and larger population of ticks^26^. Moreover, not only agricultural workers but also the general population are facing an increased risk of tick exposure, due to the rising popularity of outdoor activities such as hiking, camping, and the increasing pet population^27^. The growing risk of tick-borne diseases necessitates research on the distribution of ticks and the presence of the pathogens they carry. Therefore, we investigated the distribution of ticks and tick-borne pathogens in Gwangju city, located in southwestern region of Korea, from 2019 to 2022.

## Results

### Distribution of field collected ticks

**Figure 1:**
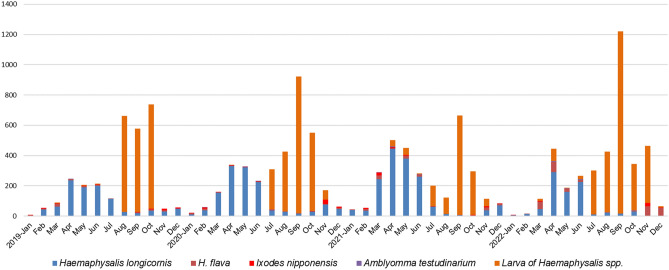
Seasonal distribution of tick samples collected in Gwangju, Korea.

**Figure 2:**
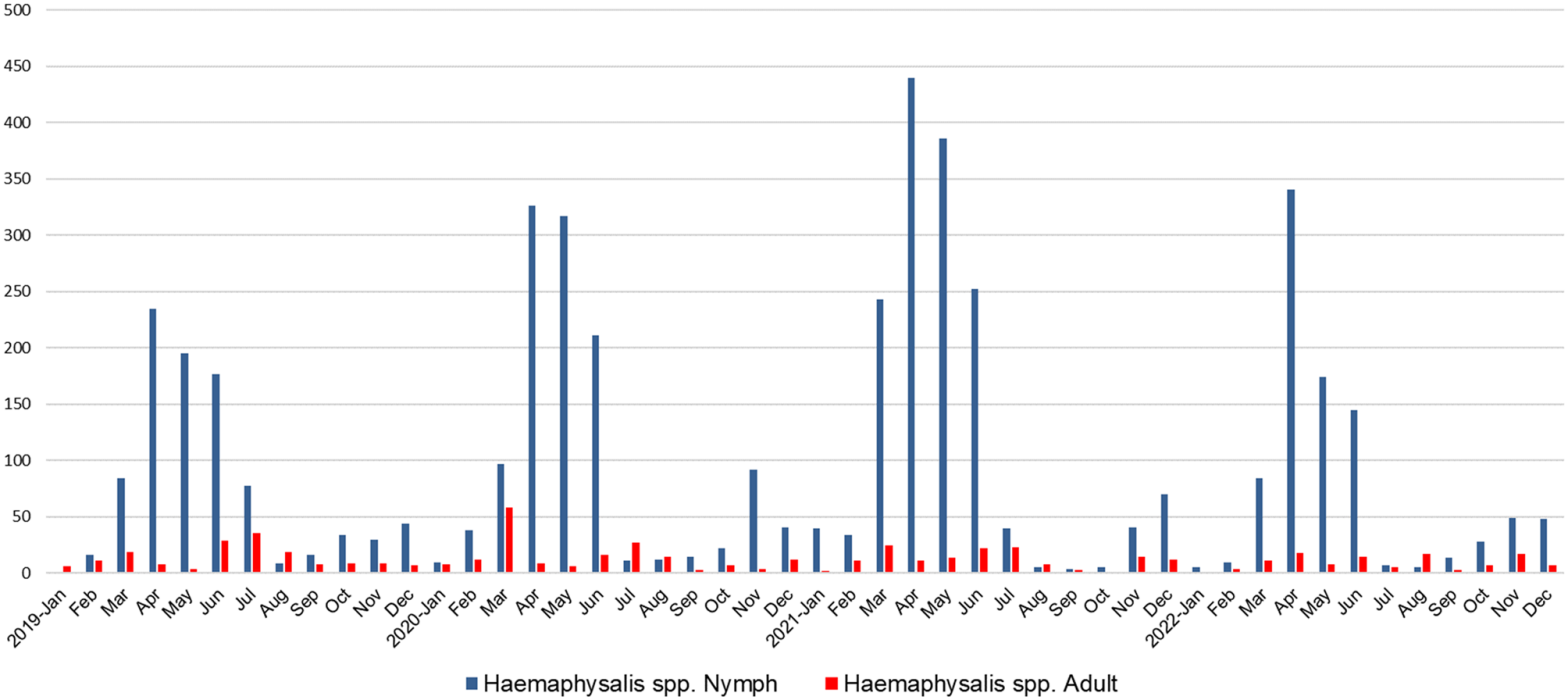
Seasonal distribution of *Haemaphysalis* species per life stage (adults and nymph) collected in Gwangju, Korea.

**Figure 3:**
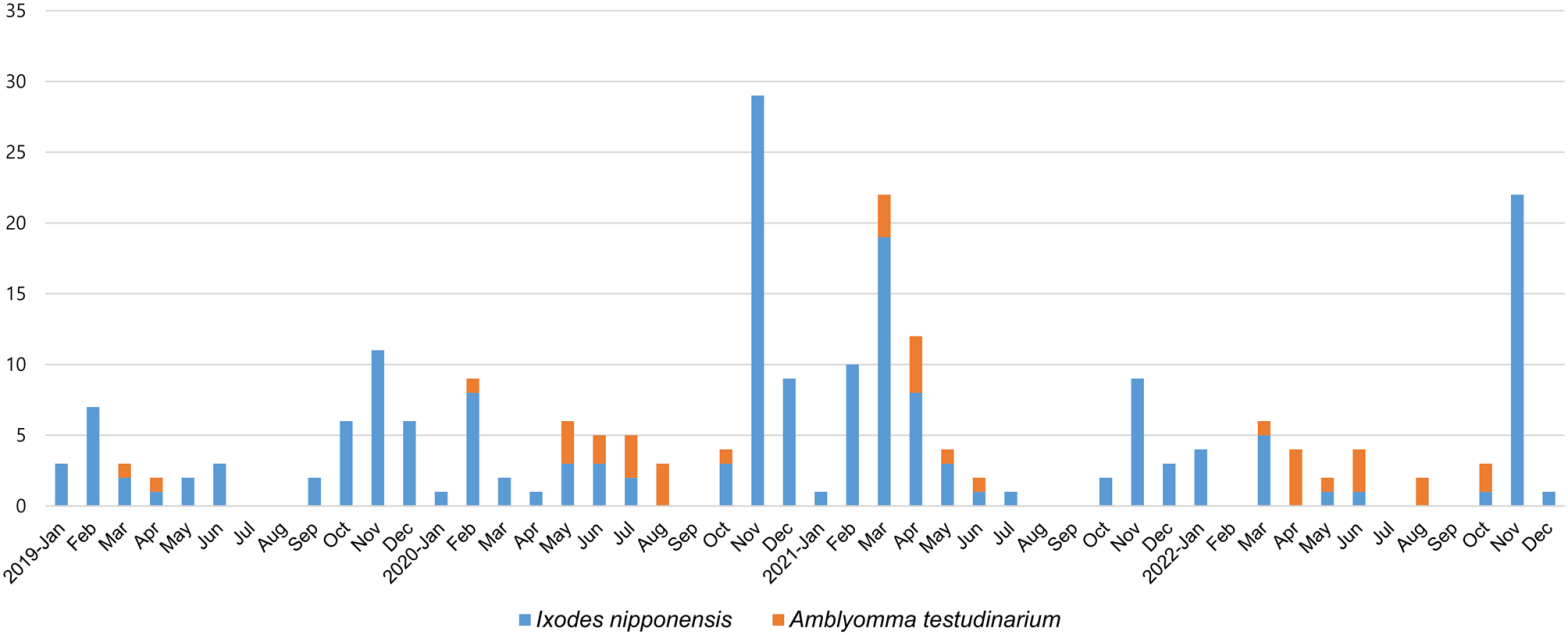
Seasonal distribution of *Ixodes nipponensis* and *Amblyomma testudinarum* ticks collected in Gwangju, Korea.

During the study period in Gwangju, South Korea, a total of 13,280 ticks were collected. Among the adult and nymph ticks, *H. longicornis* accounted for 86.1% of the collected ticks, with 349 adults and 4320 nymphs. *H. flava* comprised 9.4% of the ticks, with 252 adults and 260 nymphs. *I. nipponensis* made up 3.6% of the ticks, with 169 adults and 27 nymphs. *A. testudinarium* constituted 0.8% of the ticks, with 3 adults and 42 nymphs. Additionally, there were 7858 larvae that were difficult to differentiate between *H. longicornis* and *H. flava*, accounting for 59.2% of the total ticks collected, as shown in Table 1. Ticks were collected throughout the year, with the highest prevalence observed in the spring and fall seasons, as illustrated in Figs. 1 and 2. Specifically, larvae were primarily collected in the spring, while nymphs were predominantly collected in the fall. Interestingly, *Ixodes* ticks were observed from autumn to spring, while *A. testudinarum* ticks were observed in early summer (Fig. 3).

**Table 1:** Prevalence (minimum infection rate, %) of pathogens in field-collected ticks in Korea, 2019–2022.

Pathogen species	Tick species																	
	*Haemaphysalis longicornis*				*Haemaphysalis flava*				*Ixodes nipponensis*				*Amblyomma testudinarium*				Larvae of haemaphysalis. spp.	All ticks
	Total	Male	Female	Nymph	Total	Male	Female	Nymph	Total	Male	Female	Nymph	Total	Male	Female	Nymph		
*Total no. of ticks*	4669	40	309	4320	512	110	142	260	196	87	82	27	45	3	0	42	7858	13,280
*Total no. of pools*	460	29	151	280	194	78	89	27	135	57	56	22	37	3	0	34	157	983
*Borrelia* total	1 (0.02)			1 (0.02)					21 (13.8)	11 (12.6)	15 (18.3)	1 (3.7)						28 (0.21)
*B. afzelii*									21 (10.7)	10 (11.5)	10 (12.2)	1 (3.7)						21 (0.16)
*B. garinii*									2 (1.02)		2 (2.44)							2 (0.02)
*B. Miyamotoi*									2 (1.02)		2 (2.44)							2 (0.02)
B. Spp.*	1 (0.02)			1 (0.02)					2 (1.02)	1 (1.15)	1 (1.22)							2 (0.02)
*A. phagocytophilum*	11 (0.24)		1 (0.3)	10 (0.23)					4 (2.04)	1 (1.15)	3 (3.66)							15 (0.13)
*Ehrlichia* spp.	3 (0.06)		1 (0.3)	2 (0.05)														3 (0.02)
*Rickettsia* total	102 (2.18)	10 (25)	19 (6.1)	73 (1.69)	11 (2.15)	5 (4.55)	3 (2.11)	3 (1.15)	27 (13.8)	13 (14.9)	12 (14.6)	2 (7.41)	6 (13.3)		–	6 (14.3)	70 (0.89)	216 (1.63)
*R. canadensis*	4 (0.09)			4 (0.09)									1 (2.22)		–	1 (2.38)		5 (0.04)
*R. japonica*	91 (1.95)	10 (25)	17 (5.5)	64 (1.48)	8 (1.56)	2 (1.82)	3 (2.11)	3 (1.15)	1 (0.51)	1 (1.15)					–		60 (0.76)	160 (1.2)
*R. monacensis*	1 (0.02)			1 (0.02)	1 (0.2)	1 (0.91)			22 (11.2)	9 (10.3)	11 (13.4)	2 (7.41)	5 (11.1)		–	5 (11.9)	2 (0.03)	31 (0.23)
R. spp. *	6 (0.13)		2 (0.6)	4 (0.09)	2 (0.39)	2 (1.82)			4 (2.04)	3 (3.45)	1 (1.22)				–		8 (0.1)	20 (0.15)
*Total*	117 (2.51)	10 (25)	21 (6.8)	86 (1.99)	11 (2.15)	5 (4.55)	3 (2.11)	3 (1.15)	58 (29.6)	25 (28.7)	30 (36.6)	3 (11.1)	6 (13.3)		–	6 (14.3)	70 (0.89)	262 (1.97)

*The PCR targeting a specific gene did not reveal any distinct species as the sequencing quality of the samples was too low

### Detection of tick-borne pathogens

A total of 983 pools were tested, including 460 pools for *H. longicornis*, 194 pools for *H. flava*, 135 pools for *I. nipponensis*, 37 pools for *A. testudinarium*, and 157 pools for larvae. Adult ticks were analyzed individually or pooled, while nymphs and larvae were pooled according to their species, sex, and life stage to compare the prevalence of pathogens. In total, 2.0% (minimum infection rate, MIR) of the field-collected ticks were found to be pathogen-positive; *H. longicornis, H. flava, I. nipponensis, A. testudinarium*, and larvae (*Haemaphysalis* spp.) exhibited detection rates of 2.5%, 2.2%, 29.6%, 13.3%, and 0.9% MIR, respectively. Compared to other tick species, *I. nipponensis* and *A. testudinarium* demonstrated significantly higher pathogen prevalence rates (P < 0.001). Specifically, *I. nipponensis* exhibited a high detection rate for both *Borrelia* spp. and *Rickettsia* spp., while *A. testudinarium* was mainly associated with *Rickettsia* spp.

*Rickettsia* spp. were the most prevalent pathogens across all tick species (216 pools, 1.6% MIR, P < 0.001). Notably, *Rickettsia* spp. exhibited a high MIR in both *I. nipponensis* (13.8% MIR, P < 0.001) and *A. testudinarium* (13.3% MIR, P < 0.001).

*Borrelia* spp. were the second most commonly reported pathogen in this study (28 pools, 0.2% MIR). It was mainly detected in *I. nipponensis* (27 pools, 13.8% MIR, P < 0.001), with only one positive pool found in *H. longicornis* ticks.

*A. phagocytophilum* (15 pools, 0.1% MIR) was found in *H. longicornis* (11 pools, 0.2% MIR) and *I. nipponensis* (4 pools, 2.0% MIR), while *Ehrlichia* spp. (3 pools, 0.02% MIR) were confirmed only in *H. longicornis* ticks.

Co-infections were detected in *I. nipponensis* (14 pools) and larvae of *Haemaphysalis* spp. (6 pools); *Borrelia* spp*.* and *Rickettsia* spp. were found in 12 pools of *I. nipponensis*; *Borrelia* spp*.* and *A. phagocytophilum* were found in 1 pool of *I. nipponensis*; *Rickettsia* spp. and *A. phagocytophilum* were found in 6 pool of larvae (*Haemaphysalis* spp.). Additionally, one female *I. nipponensis* tick was positive for 3 pathogens simultaneously; *Borrelia* spp*.* and *Rickettsia* spp. and *A. phagocytophilum*. Meanwhile, there were no positive samples for SFTSV and *Coxiella burnetii* in this study.

### Molecular and phylogenetic analysis

**Figure 4:**
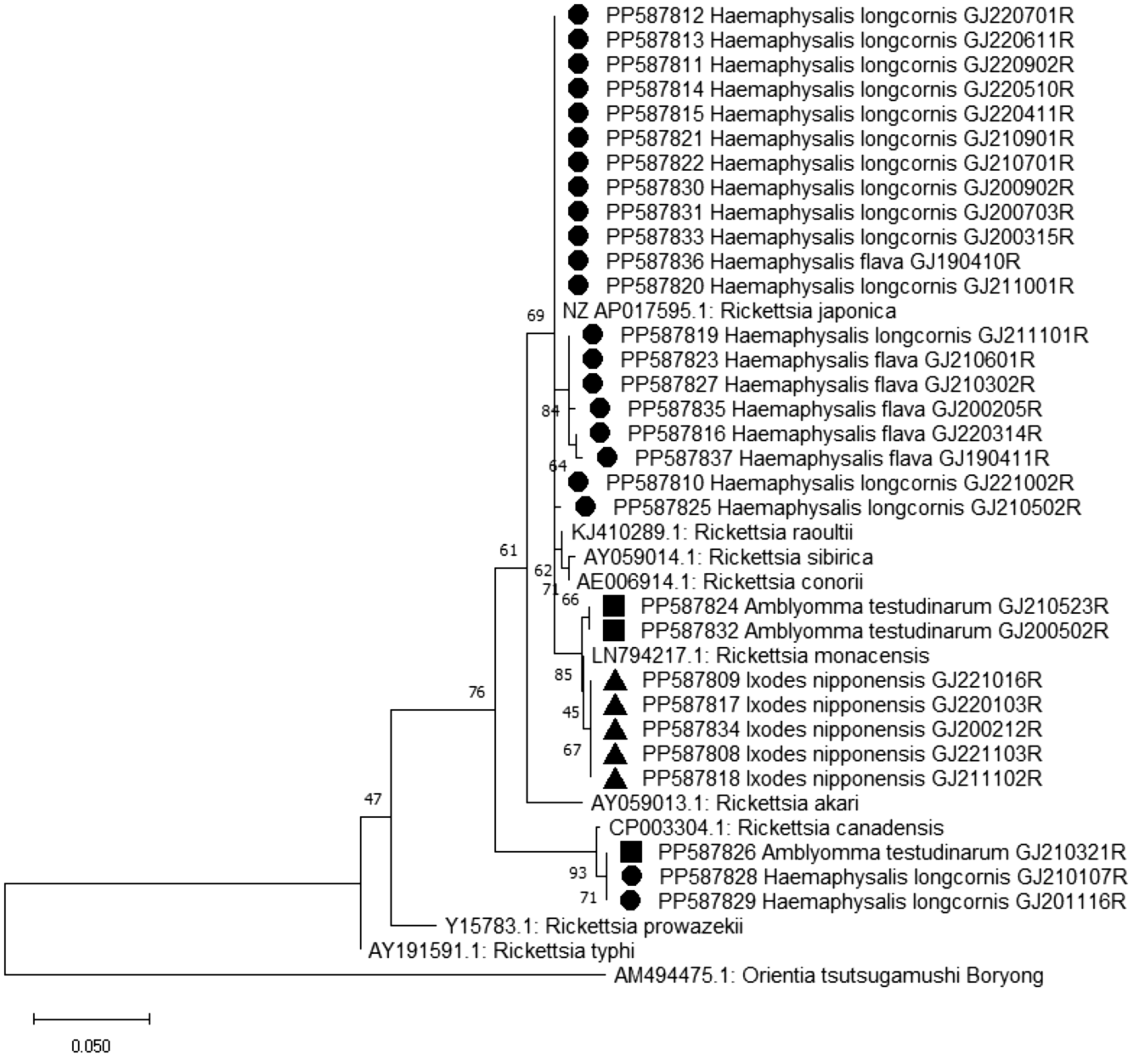
Phylogenetic relationship for *Rickettsia* species, based on the nucleotide sequences of *gro*EL gene. The neighbor-joining method was used for constructing a phylogenetic tree. Sequences identified in this study are indicated by black circles (●) for *Haemaphysalis* spp, black triangles (▲) for *I. nipponensis*, and black squares (■) for *A. testudinarum*. Scale bar indicates sequence distances.

Sequencing analysis of *groEL* gene, obtained from 196 *Rickettsia* spp. positive samples (196/612), revealed the presence of *R. canadensis, R. japonica*, and *R. monacensis* in 5, 160, and 31 pools, respectively. Among the *Rickettsia* species, *R. japonica*, the etiological agent of Japanese spotted fever (JSF), exhibited the highest detection frequency, with 160 pools (1.2% MIR) of all ticks testing positive. The majority of sequences of *R. japonica* were detected in *Haemaphysalis* spp (159/160 pools); only one adult *I. nipponensis* tick tested positive for this pathogen. Interestingly, *R. japonica* was predominantly detected in male *H. longicornis* ticks (25% MIR, P < 0.001). The nucleotide sequences of *R. japonica* showed significant similarity to those identified in humans from Japan (AP017595) (Fig. 4). *R. monacensis* was the second most frequently detected species. Unlike *R. japonica*, which was primarily detected in *Haemaphysalis* spp., *R monacensis* exhibited a remarkably high prevalence in *I. nipponensis* and *A. testudinarium* ticks. The nucleotide sequences of *R. monacensis* showed close similarity to those obtained from *Ixodes ricinus* ticks collected in Munich, Germany (LN794217) (Fig. 4). *R. canadensis*, recently recognized as a pathogenic species^46^, was detected in 4 pools of *H. longicornis* nymphs and in one pool of *A. testudinarium* nymphs.

**Figure 5:**
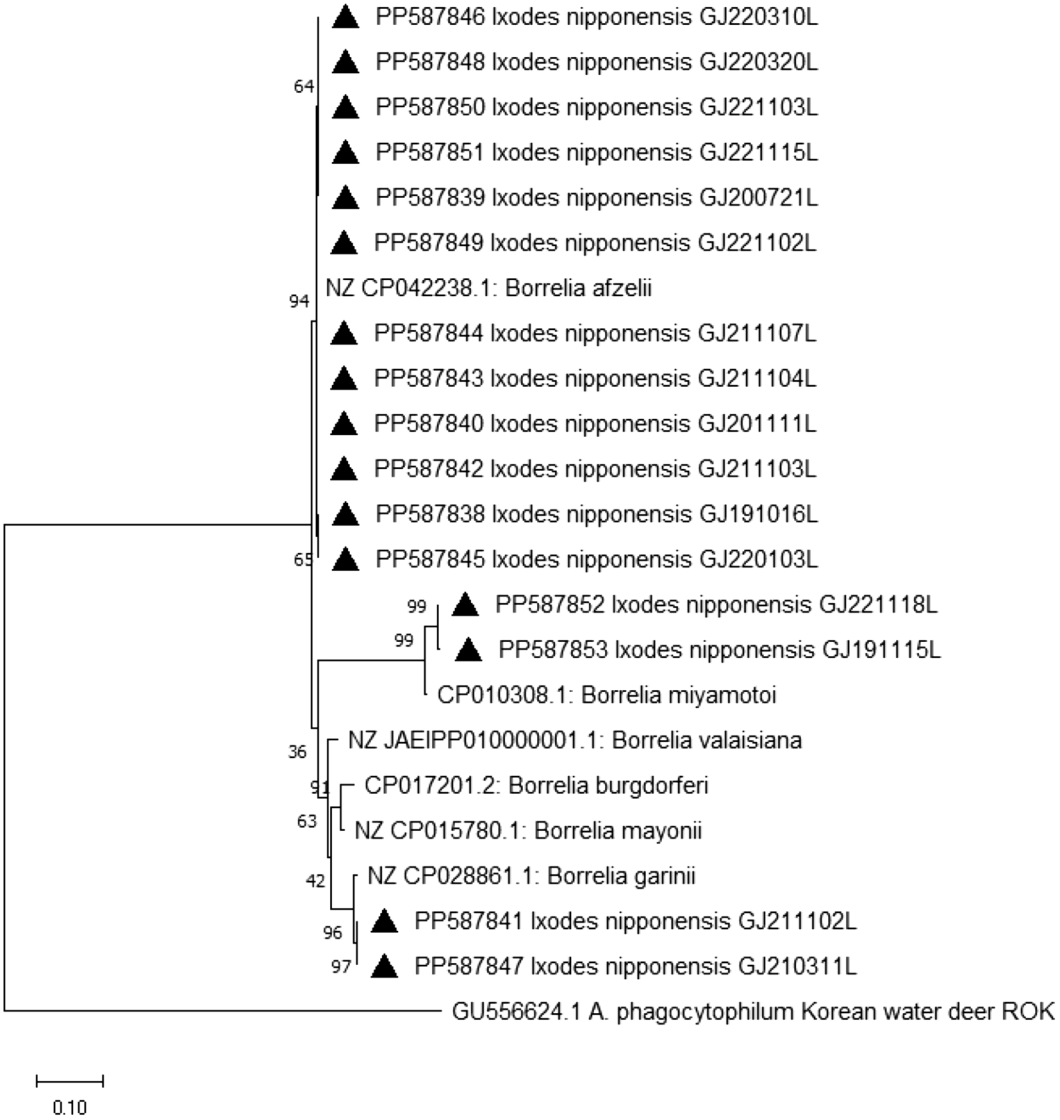
Phylogenetic relationship for *Borrelia* species, based on the nucleotide sequences of *fla*B gene. The neighbor-joining method was used for constructing a phylogenetic tree. Sequences identified in this study are indicated by black triangles (▲). Scale bar indicates sequence distances.

*Borrelia* spp., the causative agents of Lyme disease and tick-borne relapsing fever, were predominantly detected in *I. nipponensis* ticks (13.8% MIR), and in one pool of *H. longicornis* ticks (Fig. 5). Nucleotide sequence analysis revealed the presence of *B. afzelii* in 20 adult pools and 1 pool from nymphs of *I. nipponensis* ticks. The partial *flaB* sequences of the *B. afzelii* group showed high identity with that of *B. afzelii* detected in *I. nipponensis* tick in Korea (MH102391). *Borrelia garinii* and *Borrelia miyamotoi* were each detected in two pools from female *I. nipponensis* ticks, respectively.

**Figure 6:**
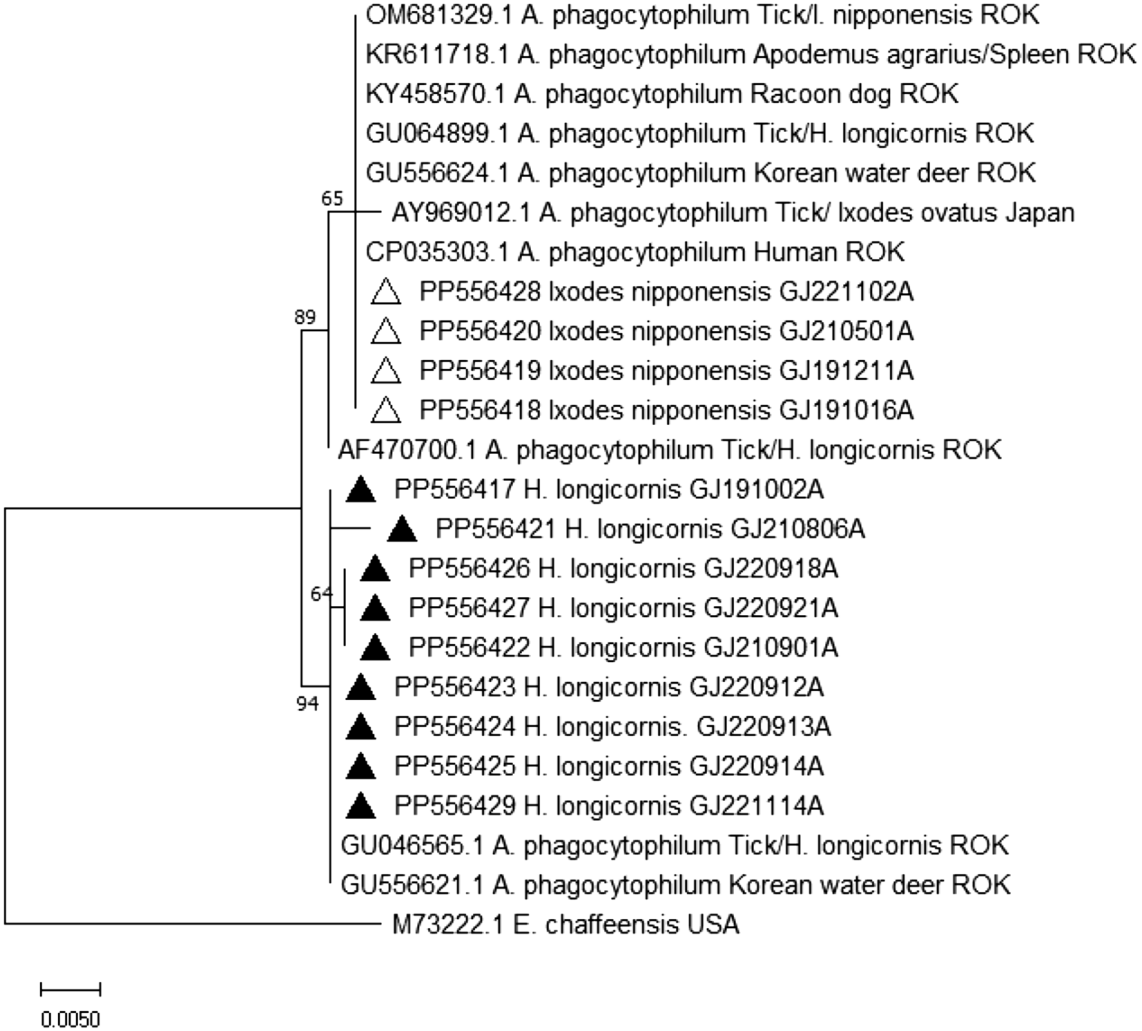
Phylogenetic relationship for *A. phagocytophilum*, based on the nucleotide sequences of 16 s rRNA gene. The neighbor-joining method was used for constructing a phylogenetic tree. Sequences identified in this study are indicated by black triangles (▲) for *Haemaphysalis* spp, and empty triangles (△) for *I. nipponensis*. Scale bar indicates sequence distances.

*A. phagocytophilum* was detected in *I. nipponensis* ticks (2.0% MIR) and *H. longicornis* ticks (0.24% MIR). Considering the sample size, the detection rate was notably high in *I. nipponensis* ticks. The nucleotide sequences displayed high similarity to those reported in Korea (OM681329). Additionally, 10 pools from *Haemaphysalis* spp. nymphs exhibited nucleotide sequences highly similar to those found in deer and ticks in Korea (GU046565, GU556621) (Fig. 6).

**Figure 7:**
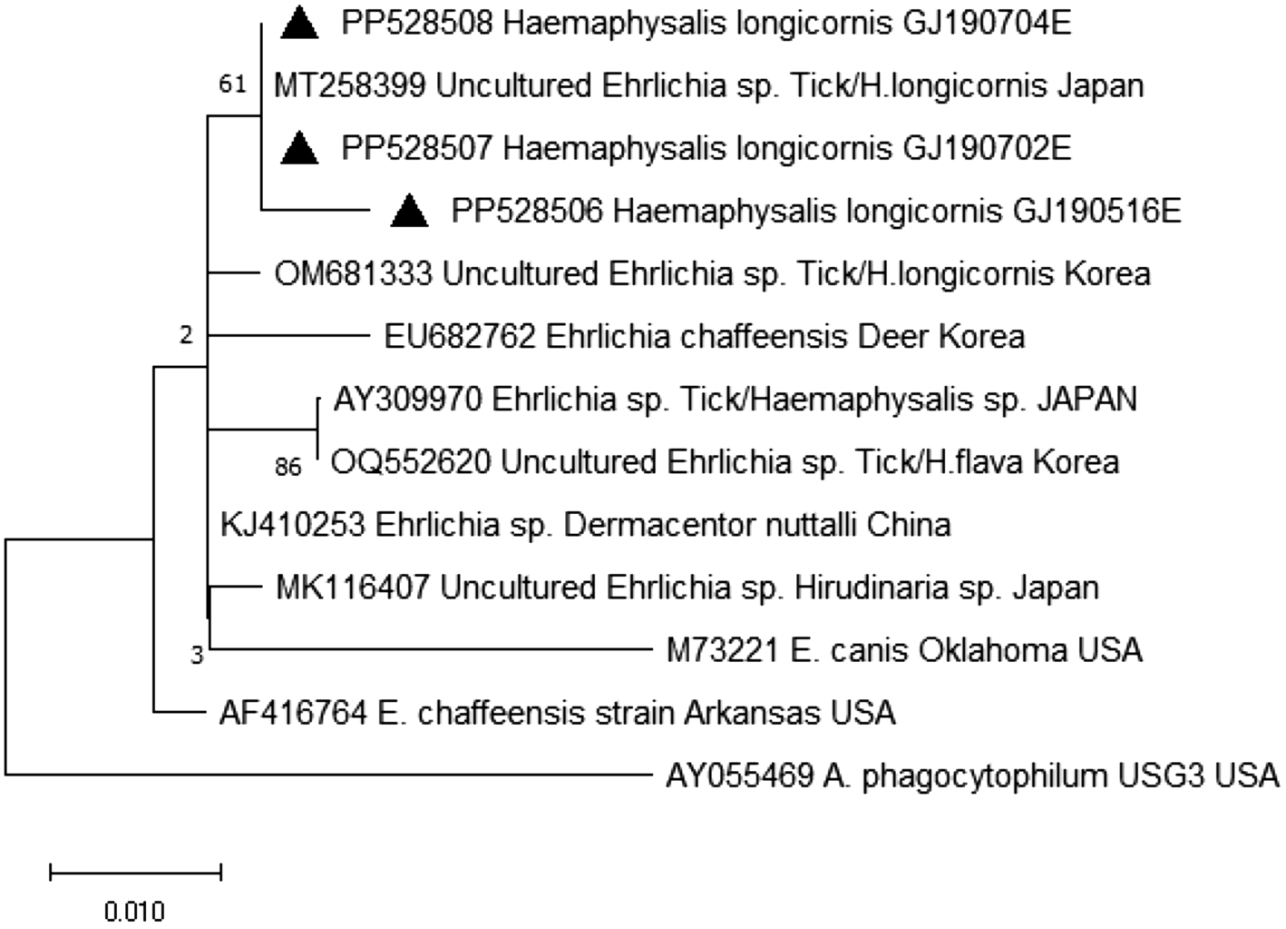
Phylogenetic relationship for *Ehrlichia* species, based on the nucleotide sequences of 16s rRNA gene. The maximum likelihood method was used for constructing a phylogenetic tree. Sequences identified in this study are indicated by black triangles (▲) for *Haemaphysalis* spp. Scale bar indicates sequence distances.

*Ehrlichia* spp. were exclusively detected in *H. longicornis* ticks; however, the PCR targeting the 16S rRNA gene did not reveal any distinct *Ehrlichia* species (Fig. 7).

In our study, 0.25% MIR of the field-collected ticks were found positive for more than one tick-borne pathogen, primarily in *I. nipponensis* ticks and larvae of *Haemaphysalis* spp. *R. monacensis* and *B. afzelii* were identified in *I. nipponensis* ticks (MIR 6.5%); *R. monacensis* and *B. garinii* in *I. nipponensis* ticks (MIR 1.2%); *B. afzelii* and *A. phagocytophilum* in *I. nipponensis* ticks (MIR 1.2%). Both *R. japonica* and *A. phagocytophilum* were detected in 6 pools from larvae (MIR 0.1%). Furthermore, three pathogens were identified in one pool of *I. nipponensis*; *R. monacensis, B.afzelli*, and *A. phagocytophilum*.

## Discussion

In South Korea, the incidence rate of tick-borne diseases mediated by hard ticks is relatively low compared to countries such as the United States and Europe. For instance, the reported occurrence of Lyme disease in the United States is 73.3 cases per 100,000 individuals^28^, while in Finland, it is around 118 cases^29^. Canada reported 7.0 cases in 2019^30^, and Lyme disease patients are reported annually in China and Japan as well^31^. In Korea, the total number of confirmed cases over a 10-year period from 2012 to 2021 was exceptionally low, with only 110 cases (domestically)^32^. Consequently, research on these ticks, which serve as vectors for TBPs, is limited. However, there is an increasing possibility of a higher incidence of tick-borne diseases due to the introduction of exotic tick species and the potential introduction of pathogens facilitated by climate change^33,34^. Factors such as increased international exchanges and outdoor activities contribute to this possibility^34^. This study aimed to investigate the distribution of tick species, seasonal variations, and conduct molecular epidemiological analysis of pathogens in field ticks from the southwestern region of Korea between 2019 and 2022.

During the study period, a total of 13,280 hard ticks were collected, and the distribution of ticks was in accordance with other studies conducted in Korea. According to research by Seo et al*.*^35^, which utilized the dry-ice trap method throughout Korea, out of 63,376 hard ticks collected, *H. longicornis* accounted for 96.5%, *H. flava* for 2.8%, *I. nipponensis* for 1.7%, and *A. testudinarium* for 0.5%. In a study conducted by Lee et al*.*^3^, utilizing the flagging and dragging method, *H. longicornis* accounted for 80.7%, and *H. flava* accounted for 16.2%. Similarly, in this study, *H. longicornis* was identified as the dominant species, accounting for 86.1%. *A. testudinarium* were found very rarely. *A. testudinarium* was mainly found in southern region of Korea^3^.

In Korea, adult ticks and nymphs typically peak from May to August, while larvae peak from August to September^2,36,37^. Following their life cycle, adult ticks lay eggs during the summer, which then hatch into larvae. These larvae predominantly feed on hosts in the autumn and subsequently molt into nymphs, which spend the winter to spring period. Consequently, there is a higher distribution of nymphs in the spring and larvae in the autumn^35^. In this study, adult ticks and nymphs were primarily collected from April to July, while larvae were collected from July to November. Notably, *Ixodes* ticks were predominantly collected from autumn to spring in this study. Since there have been no previous reports on the distribution of *I. nipponensis* ticks, including the winter season, in Korea, this observation represents a novel finding. It underscores the importance of continuous monitoring of *I. nipponensis* tick distribution throughout Korea.

Meanwhile, there are reports suggesting that climate change can affect tick abundance^38,39^. When the seasons favored by nymphs and larvae overlap, there is an increased risk of pathogen transmission as they can co-feed on the same host. In cases of tick-borne pathogens with non-systemic infection, larvae have no chance of acquiring the pathogen. However, with climate change leading to simultaneous population peaks of nymphs and larvae, infected nymphs can transmit the pathogen to larvae via the host's blood system, without causing systemic infection^38,39^. The results of this study also indicate a trend of increasing ambiguity in the timing of peak periods for adult ticks, nymphs, and larvae after 2019. This highlights the need for continuous monitoring of tick distribution and its peak seasons. It should be noted that factors such as the specific method employed for collecting ticks, the timing of the collection, and the type of sampler utilized can introduce some degree of variability in species distribution and seasonal patterns.

The results confirmed that the detection rate of *Rickettsia* spp. was the highest when examining the pathogens carried by field ticks in southwestern Korea. This result may reflect the characteristics of the pathogen, such as trans-stadial and trans-ovarial infections in ticks^40^. Similar findings were reported in studies conducted in Latvia^41^, Spain^42^, and France^43^, where the detection rate of *Rickettsia* spp. was found to be the highest. On the other hand, in a study conducted in Finland, *Borrelia burgdorferi*, the causative agent of Lyme disease, was predominantly detected^44^. Meanwhile, information about the prevalence of various tick-borne pathogens, including *Rickettsia* spp. in Korea is very scarce. Our report confirms the potential risk of *Rickettsia* spp. to humans.

In the Asian region, *Rickettsia* spp. have been predominantly detected in *Haemaphysalis* spp., *A. testudinarium*, or *Dermacentor* spp. Among *Rickettsia* species, *R. japonica* is commonly reported to be confirmed mainly in *Haemaphysalis* spp. or *Dermacentor* spp., while *R. monacensis* has been reported in *Amblyomma dissimile*, *Dermacentor variabilis, Ixodes boliviensis, I. persulcatus, I. ricinus, I. sinensis*, *I. pacificus*, and *Rhipicephalus sanguineus*^45–47^. In this study, *R. japonica* was predominantly confirmed in *Haemaphysalis* spp., which is consistent with previous findings^45,46^.

*R. monacensis* was detected at a very high rate in *I. nipponensis.* A study was conducted on ticks collected from wild rodents captured in the U.S. military bases and training grounds located in Korea, where *I. nipponensis* accounted for 99.5% of the ticks collected. Among the 197 pooled samples, *Rickettsia* spp. was detected in 58.4%, with 87 pools of *R. monacensis* and 18 pools of *R. japonica*^48^. Recent studies conducted in Korea and Japan also reported a high correlation between *R. monacensis* and *I. nipponensis*^49,50^.

Interestingly, *R. monacensis*, known to cause a Mediterranean spotted fever-like illness^51,52^, was detected at a very high rate in *A. testudinarium,* as well. To the best of our knowledge, this study represents the first report of this pathogen being detected in *A. testudinarium*. In a previous study conducted from 2014 to 2018 in Korea^4^, no pathogens were detected in *A. testudinarium*. *A. testudinarium* is primalrily reported to inhabit southern regions of Korea, and it is necessary to monitor whether their habitats are changing due to climate change.

*Ixodes* ticks are recognized as major vectors of *Borrelia* bacteria, with *I. persulcatus* or *I. nipponensis* being identified in Asia. In a study of ticks collected from pasture around livestock farms in Korea, the detection rate of *Borrelia* spp. was found to be 34.0% MIR in *I. nipponensis*^3^. Another study in Korea also reported that the MIR of *Borrelia* spp. in *I. nipponensis* was 2.1%, while in *H. longicornis* and *H. flava*, it was 0.09%, and 0.1%, respectively^53^. The incidence of Lyme disease is associated with *Borrelia* genotypes, yet there are few reports on genotypes in field ticks in Korea. *B. afzelii* and *B. miyamotoi* were previously detected in 3 pools (12.0%), and 1 pool (4%), respectively in *I. nipponensis* adults in Korea^54^. Lee et al*.* reported that all *Borrelia* species, detected in *I. nipponensis* collected from Korean water deer and by tick drag were identified as *B. afzelii* by the *ospA* gene sequences^55^. During our survey, *B. afzelii, B.garinii,* and *B. miyamotoi* were detected in 10.7%, 1.0%, and 1.0%, respectively. These results indicate *B. afzelii* is common, while *B.garinii,* and *B. miyamotoi* are rarely detected in *I. nipponensis* in Korea.

*A. phagocytophilum* is reported to be transmitted by *Ixodes* ticks. In the United States, representative vectors include *Ixodes scapularis* or *Ixodes pacificus*. In Western Europe, transmission is primarily associated with *I. ricinus*, while in Asia, *I. persulcatus* is known as a vector^56^. In this study, *I. nipponensis* exhibited high detection rates of *A. phagocytophilum*.

Research on the genus *Ehrlichia* has been limited in Korea. In a study investigating TBPs in ticks from grazing cattle in Korea, *E. canis* was detected with the highest rate, followed by *E. chaffeensis, E. ewingii*, and *E. muris*^57^. Another study found *E. chaffeensis* was the most frequently detected species in *H. longicornis* ticks collected in northern Korea^58^. However, in this study, the PCR targeting the 16S rRNA gene did not reveal any distinct *Ehrlichia* species. Three samples belonging to the genus *Ehrlichia* clustered with *Ehrlichia* spp. detected in *H. longcornis* in Japan (MT258399) . Since the 16S rRNA gene is known to be more conserved, further study is warranted by comparing more divergent gene sequences, such as the *groEL* gene.

*Ixodes* ticks are commonly co-infected with other tick-borne pathogens such as *Borrelia* spp., *Babesia* spp., *Ehrlichia* spp., *Rickettsia* spp., and Powassan virus^59^. In this study, co-infections were observed in a total of 14 pools of *I. nipponensis*, mostly consisting of *Borrelia* spp. and *Rickettsia* spp. These findings corroborate previous research suggesting that co-infection is common among *Ixodes* ticks^41,59^. Co-infection may lead to increased diversity, severity, and duration of symptoms^60^. Therefore, raising awareness of potential co-infections is crucial, and further studies are warranted.

Meanwhile, *Coxiella burnetii* was not detected in this study. Its primary route of transmission is through the inhalation of contaminated aerosols^18^. Although *C. burnetii* has been detected in various tick species, tick-borne transmission is considered to be low^19^. A case report described an 8-year-old Korean girl who was co-infected with the SFTS virus and *C. burnetii* after playing with a dog and being bitten by a tick. In a study of 816 horses in Korea, six samples (0.7%) tested positive by PCR, suggesting the potential for *C burnetii* transmission to humans during horseback riding. Additionally, other studies provided evidence of ticks harboring *C burnetii* in Korea^61,62^. Given these reports, further investigation into the epidemiology of Q fever is required.

There are several limitations to this study. Firstly, our research was focused solely on the Gwangju city, southwestern Korea, which may not be fully representative of the entire tick population in Korea. Secondly, our tick collection methods primarily relied on dragging and flagging, which could introduce sampling bias. /these method might not accurately capture the overall tick population and their habitats. Thirdly, our pooling strategy for tick samples may raise question about accuracy. Some research has shown that pools with more than 20 nymphs did not significantly improve the detection probability of *Rickettsia* species^63^. Additionally, the calculation of the minimum infection rate (MIR) assumes that only one infected individual exists in a positive pool, potentially underestimating the actual prevalence of infection. Therefore, our reported prevalence rates may represent a lower bound of the infection rate^64^. Lastly, our analysis of *Rickettsia* species was based on the *groEL* gene. While this gene provides valuable information, a more comprehensive analysis could have been achieved by analyzing additional sequences targeting genes such as *rrs, gltA*, and *ompA*. Despite these limitations, our study offers valuable insight into the distribution and pathogen characteristics of hard ticks in the natural environments of Korea. This is particularly relevant for less-studied species like *I. nipponensis* and *A. testudinarium*, which have been underrepresented in research due to their smaller collection scale compared to other tick species. Future research incorporating a broader range of molecular markers and expanding the geographic scope of sampling could further enhance our understanding of tick-borne diseases in Korea.

In conclusion, this study comprehensively investigated the distribution of hard ticks and characteristics of TBPs using 13,280 specimens collected from the southwestern region of Korea between 2019 and 2022. *Rickettsia* spp. was the most commonly detected pathogen, with *R. japonica* and *R. monacensis* being frequently detected in *I. nipponensis* and *Haemaphysalis* spp., respectively. Notably, our study identified *R. monacensis* for the first time in *A. testudinarium* in South Korea. These findings underscore the imperative for continuous research on indigenous hard ticks and associated pathogens in South Korea. Given the impact of factors such as climate change, increased international exchange, and alterations in wildlife behavior on the dynamics of tick-borne diseases, continuous monitoring is essential to detect and manage the potential introduction of exotic ticks and pathogens, which could pose new challenges for human health.

## Materials and methods

### Tick sampling and classification

Ticks were collected from the field on a monthly basis for 1–2 days by using the dragging and flagging methods. A 1 m × 1 m white flannel cloth attached to a wooden bar was utilized for this purpose. The collection was conducted in Gwangju city, situated in the southwestern region of the Republic of Korea. Collection sites comprised 3–5 locations in hills and mountainous areas surrounding Gwangju city, chosen based on accessibility or suitablility for crop cultivation. The collection process involved three to four collectors, each spending 15–20 min. Ticks were carefully removed from the flannel cloth using fine forceps and transferred to 50 mL tubes. Subsequently, the collected ticks were stored at − 80 °C until further processing. Identification of ticks was performed using an Axio Zoom.V16 microscope, following the guidelines provided by Yamaguti et al*.*^65^.

### DNA/RNA extraction

Ticks were pooled in Precellys® 2 mL tubes along with 2 mm ceramic bead and 700 μL of sterile phosphate-buffered saline. Depending on the species, sex, collection date, and stage of development, 1–2 adults, 1–30 nymphs, and 1–50 larvae were pooled from each collection site for comparison in the prevalence of pathogens. The pooled ticks were homogenized for 1 min at 8000 rpm using Precellys® 2000 homogenizer and then centrifuged for 5 min at 10,000 rpm. The resulting supernatants were subjected to DNA/RNA extractions using the Maxwell® RSC viral total nucleic acid purification kit (Promega, Wisconsin, USA), following the manufacturer’s instruction.

### Detection and characterization of pathogens

The nucleic acids were analyzed using the Applied Biosystems QuantStudio™ 5 real-time polymerase chain reaction (qPCR) machines, while the remaining samples were stored at 4℃ for further analysis. Molecular identification of SFTSV, *Rickettsia spp., C. burnetii, Borrelia spp., A. phagocytophilum,* and *Ehrlichia* spp. was carried out using qPCR assays with Popgen® pathogen detection kits (PostBio, Gyeonggi-do, Korea), following the manufacturer’s instructions. Briefly, the PCR assay was performed in 20 μL reaction mixtures consisting of 15 μL of Popgen® qPCR reaction Mix (aliquot) and 5 μL of template DNA. The reaction conditions included an initial denaturation step at 95 °C for 5 min, followed by 45cycles of denaturation at 95 °C for 10 s and annealing/extension at 60 °C for 30 s.

Any positive pools identified in the screening assay were subsequently confirmed by the ProFlex™ PCR machine using primer sets as listed in Table 2^66–73^. The amplified PCR products were sent to Bionics (Daejeon, Korea), for sequencing using an ABI 3730XL DNA Analyzer (Applied Biosystems, Foster City, USA). The nucleotide sequences obtained were aligned using ClustalW within MEGA-X software and compared with GenBank database using the Basic Local Alignment Search Tool (BLAST) at the National Center for Biotechnology Information (NCBI).

**Table 2:** Primers for the detection of tick-borne pathogens.

Pathogens	Primer	Nucleotide sequence (5′–3′)	Product size	References
SFTSV (M segments)	SFTS_M_F1	TCATCCTGACTATTYAGCAATWG	640	^66^
	SFTS_M_R2	TAAGTYACACTCACACCCTTGAA		
	MF3	GATGAGATGGTCCATGCTGATTCTAA	560	^67^
	MR2	CTCATGGGGTGGAATGTCCTCAC		
*Anaplasma* and *Ehrlichia* spp.	AE1-F	AAGCTTAACACATGCAAGTCGAA	1406	^68^
	AE1-R	AGTCACTGACCCAACCTTAAATG		
*Anaplasma* spp. (16S rRNA)	AP-F	GTCGAACGGATTATTCTTTATAGCTTGC	926	^69^
	AP-R	CCCTTCCGTTAAGAAGGATCTAATCTCC		
*Ehrlichia* spp. (16S rRNA)	EC-F	CAATTGCTTATAACCTTTTGGTTATAAAT	390	
	EC-R	TATAGGTACCGTCATTATCTTCCCTAT		
*Borrelia* spp. (*flaB*)	132-F	TGGTATGGGAGTTTCTGG	774	^70^
	905-R	TCTGTCATTGTAGCATCTTT		
	220-F	CAGACAACAGAGGGAAAT	605	
	824-R	TCAAGTCTATTTTGGAAAGCACC		
*Coxiella* spp. (*htpAB*)	IS111-F1	TACTGGGTGTTGATATTGC	485	^71^
	IS111-R1	CCGTTTCATCCGCGGTG		
	IS111-F2	GTAAAGTGATCTACACGA	260	^72^
	IS111-R2	TTAACAGCGCTTGAACGT		
*Rickettsia* spp. (*groEL*)	groEL OF	GTTGAAGTATGTTAAAGG	534	^73^
	groEL ON	TTTTTCTTTATTCATAATC		
	groEL OFN	GTAGTTAAAGGTATGATGTTTGATA	468	
	groEL ORN	ATCTTCAATATTTTTCTTATCACCG		

Phylogenetic analyses were conducted using MEGA-X software (v.6.4), and the phylogenetic trees were constructed using the neighbor-joining method based on the Kimura 2-parameter model, with 1000 bootstrap replicates.

The prevalence of pathogens was calculated as minimum infection rate (MIR). The MIR for pooled ticks was determined by dividing the number of positive pools by the total number of ticks. The significance of the difference in prevalence for each pathogen among species, sexual, and developmental stages was evaluated using the chi-square test in Excel 2016.

## Data Availability

All data analysed for this study are included in this article.
